# Bacterial Manipulation of Autophagic Responses in Infection and Inflammation

**DOI:** 10.3389/fimmu.2019.02821

**Published:** 2019-12-03

**Authors:** Yang Jiao, Jun Sun

**Affiliations:** Division of Gastroenterology and Hepatology, College of Medicine, University of Illinois at Chicago, Chicago, IL, United States

**Keywords:** autophagy, effectors, bacteria, LC-3, innate immunity, microbiome

## Abstract

Eukaryotes have cell-autonomous defenses against environmental stress and pathogens. Autophagy is one of the main cellular defenses against intracellular bacteria. In turn, bacteria employ diverse mechanisms to interfere with autophagy initiation and progression to avoid elimination and even to subvert autophagy for their benefit. This review aims to discuss recent findings regarding the autophagic responses regulated by bacterial effectors. Effectors manipulate autophagy at different stages by using versatile strategies, such as interfering with autophagy-initiating signaling, preventing the recognition of autophagy-involved proteins, subverting autophagy component homeostasis, manipulating the autophagy process, and impacting other biological processes. We describe the barriers for intracellular bacteria in host cells and highlight the role of autophagy in the host-microbial interactions. Understanding the mechanisms through which bacterial effectors manipulate host responses will provide new insights into therapeutic approaches for prevention and treatment of chronic inflammation and infectious diseases.

## Introduction

Autophagy, which literally means “self-eating,” is an intrinsic process of eukaryotes that delivers cytoplasmic material to lysosomes for degradation. During this process, cytoplasmic material is enclosed by phagophores. Then, the phagophores elongate to form autophagosomes that fuse with lysosomes to form autolysosomes where the cargoes are degraded (1). Autophagy is an important biological process that is involved in immune responses, embryonic development, cell death, and cellular defense (2). It is important for host responding to nutrient stress as well as eliminating intracellular pathogens. Better understanding of autophagy mechanism will allow us to develop therapeutic drugs, vaccines, and host-directed strategies for successful control of intracellular microorganisms.

There are three forms of autophagy that are commonly described: chaperone-mediated autophagy (CMA), microautophagy, and macroautophagy. Macroautophagy is then divided into non-selective and selective autophagy. Various cargoes, such as defective mitochondria, defective endoplasmic reticulum (ER), lipids, and foreign organisms, are targeted by selective autophagy for degradation. When autophagy engulfs microorganisms for clearance, this pathway is called “xenophagy,” which plays a central role in cellular defense (3).

To survive in host cells, bacteria employ multiple mechanisms to protect against cellular defenses. Effectors, one type of weapons used by bacteria, are proteins translocated from the bacterial cytoplasm to the host cell cytoplasm by a series of secretion systems (T1SS–T8SS) (4).

Bacterial effectors have the capacity to influence host cellular biological processes, including signaling pathways, tight junctions, phagocytosis, apoptosis, and autophagy (5, 6). This review will discuss the recent research advancement (<6 years) in interactions between bacterial effectors and host autophagic responses. We synoptically describe the barriers for intracellular bacteria in host cells and highlight the role of autophagy in these processes. Furthermore, we emphasize the different strategies used by bacterial effectors from secretion systems (T3SS, T44SS, T6SS, and T7SS) to manipulate autophagic responses in host cells in infection and inflammation.

## The Fate of Intracellular Bacteria in Autophagy

**Figure 1:**
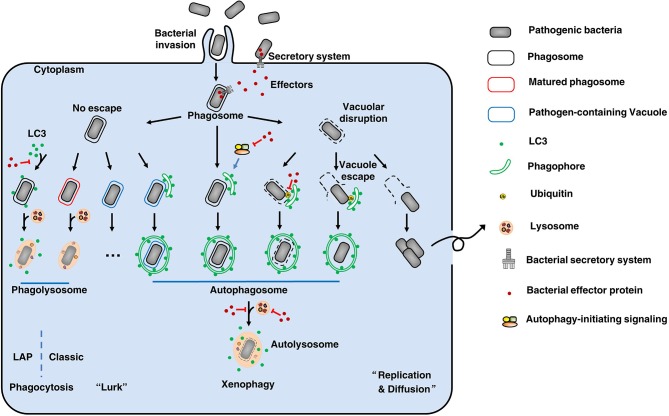
The fate of intracellular bacteria. After entry into host cells, bacteria are localized to internalization vacuoles, which are designated as phagosomes To survive, bacteria employ diverse means to escape or subvert host cellular defenses, especially using its secretion systems and effectors. By various ways, some bacteria (like *Salmonella* Typhimurium) can damage the phagosomes and then escape to the cytoplasm, where can obtain nutrients to replicate and to diffuse. On the other hand, to clean up the bacteria remaining in phagosomes, the phagosomes will be mature and fuse with lysosomes to form phagolysosomes where the bacteria are degraded. This's the classic phagocytosis. To prevent phagocytosis-mediated bacterial killing, bacteria (such as *Mycobacterium tuberculosis*) can modify the phagosomes to form pathogen-containing vacuoles, thus avoiding fusion with lysosomes. These bacteria will lurk to wait for opportunities for their survival. Therefore, xenophagy plays a key role in cell resistance to these crafty bacteria by clearing pathogen-containing vacuoles, escaped pathogens, damaged vacuoles, and pathogen-containing phagosomes. During this process, above targets are enclosed by phagophores. Then, the phagophores elongate to form autophagosomes that fuse with lysosomes to form autolysosomes where the bacteria are eliminated. Notably, LC3-associated phagocytosis (LAP) can recruit the autophagy marker protein LC3 to pathogen-containing phagosomes, and the subsequent fusion of these phagosomes with lysosomes results in pathogen digestion. Additionally, there are other unmentioned cross-talk between xenophagy and phagocytosis. Back to our theme, effectors-autophagy interactions. Using effectors delivered by secretion systems, bacteria are able to interfere with autophagy-initiating signaling, modify LC3 protein, avoid autophagosome-lysosome fusion, affect lysosome function, and deubiquitinate ubiquitinated substrate around intracellular bacteria, etc. Thus, bacteria can suppress or subvert autophagic responses for their survival. Overall, there is a constant battle between bacterial evasion mechanisms and host cellular defenses, and the fate of intracellular bacteria is determined by the outcome of this battle.

There is a constant battle between bacterial evasion mechanisms and host cellular defenses, and the fate of intracellular bacteria is determined by the outcome of this battle. Intracellular pathogens can be internalized by either phagocytic or non-phagocytic cells. After entry into host cells, bacteria are localized to internalization vacuoles, which are designated as phagosomes (Figure 1). To survive, bacterial effectors have different strategies to interfere with host, including affecting autophagy-initiating signaling, modifying LC3 protein, avoiding autophagosome-lysosome fusion, affecting lysosome function, deubiquitinating ubiquitinated substrate around intracellular bacteria, etc. Therefore, intracellular bacteria obtain nutrients to replicate or hide to wait for opportunities.

Pathogen-containing phagosomes fuse with lysosomes via phagocytosis to form phagolysosomes, where the bacteria are eliminated. Notably, recent reports have described a process called LC3-associated phagocytosis (LAP) that recruits the autophagy marker protein LC3 to pathogen-containing phagosomes, and the subsequent fusion of these phagosomes with lysosomes results in pathogen digestion (7). To prevent phagocytosis-mediated bacterial killing, bacteria can either modify the phagosomes to form pathogen-containing vacuoles, thus avoiding fusion with lysosomes (*Mycobacterium tuberculosis*), or disrupt the vacuoles to escape from the phagosomes (*Salmonella* Typhimurium) (8). Xenophagy plays a key role in cell resistance to these crafty bacteria by clearing pathogen-containing vacuoles, escaped pathogens, damaged vacuoles and pathogen-containing phagosomes. Pathogens have many unique ways to escape or subvert host xenophagy. These mechanisms are complex and fall outside the scope of this article; readers are referred to more comprehensive reviews of this subject (9, 10). Here, we focus on the effectors employed by Gram-negative bacteria to disrupt the autophagic responses of host cells.

## The Autophagy Manipulation Strategies of T3SS Effectors

Bacteria can be eliminated by autophagy, thus, Gram-negative bacteria use T3SS effectors to suppress or subvert this process. We summarized all related research work in Table 1 and discussed the recent progress (<6 years) of different strategies bacteria used, e.g., interference with signaling or ATG proteins, prevention of recognition by autophagy mechanisms, subversion of autophagic components for bacterial survival, and escape from LC3-associated phagocytosis.

**Table 1:** Strategies used by T3SS effectors to manipulate autophagy.

**Bacteria**	**Effectors**	**Host model**	**Description**	**Bacterial survival**	**References**
**Interfering with signaling or proteins involved in autophagy**					
*Shigella flexneri*	IcsB	HeLa cells, 293T cells and MEFs	Recruits the host protein Toca-1 to repress the recruitment of LC3 around these intracellular bacteria	The absence of IcsB has no effect on bacterial survival up to 3 h of infection	(11)
*Salmonella* Typhimurium	Unknown	Mouse peritoneal macrophages	Recruits FAK to SCVs and then stimulates the Akt-mTORC1 signaling pathway	In FAK-deficient macrophages, Akt/mTOR signaling is attenuated and autophagic capture of intracellular bacteria is enhanced, resulting in reduced bacterial survival	(12)
*Ralstonia solanacearum*	AWR5	Yeast, *N. benthamiana*	Suppresses TOR signaling by inhibiting TORC1 upstream of PP2A	Not applicable	(13)
*Salmonella* Typhimurium	SseF and SseG	HeLa cells, Rab1A^−/−^ RAW264.7 cell, Rab1A^−/−^ mouse	Inhibits Rab1A-mediated autophagy	SseF or SseG-deficient bacterial strains exhibit reduced survival and growth in both mammalian cell lines and mouse infection models	(14)
*Salmonella* Typhimurium	SopF	HeLa cells	Targets ATP6V0C for ADP-ribosylation on Gln124, thereby blocking recruitment of ATG16L1 by the V-ATPase	ΔsopF grow less efficiently in HeLa cells than the WT strain. And this SopF-dependent replication was diminished in ATG16L1^−/−^ cells, which were rescued by ATG16L1	(15)
*Salmonella* Enteritidis	AvrA	HCT116 cells, organoids and mice	Reduces the protein expression of Beclin-1 by inhibiting the JNK/c-Jun/AP-1 signaling pathway	AvrA-deficient bacterial strains colonized human epithelial cells show a decreased intracellular bacterial load compared to those colonized with wild type	(16)
*Burkholderia pseudomallei*	TTSS1 ATPase	RAW 264.7 cells	Decreases colocalization with LC3 but does not affect autophagy	TTSS1 ATPase-deficient bacterial strains has diminished survival and replicative capacity in RAW264.7 cells	(17)
**Preventing recognition by autophagy mechanisms**					
*Shigella flexneri*	IcsB	BHK cells, MDCK cells and *atg5*^−/−^ MEFs	Competes with ATG5 binding to the bacterial surface protein VirG	The absence of IcsB has no effect on bacterial survival up to 3 h of infection	(18)
*Shigella flexneri*	IpaH1.4	MEFs	Antagonizing the LUBAC-mediated accumulation of M1-linked ubiquitin chains on bacterial surfaces, as well as the recruitment of Optineurin and Nemo	Not applicable	(19)
*Salmonella* Typhimurium	SseL	HeLa cells, RAW264.7 cells and BMM	Splits cytosolic aggregates around SCVs by its deubiquitinating activity	SseL contributes to bacterial replication in restrictive cellular environment	(20)
**Subverting autophagic components for bacterial survival**					
*Vibrio parahaemolyticus*	VopQ	HeLa cells	Forms a gated ion channel on lysosomes	VopQ attenuates phagocytosis of *Vibrio parahaemolyticus* during infection	(21–23)
*Salmonella* Typhimurium	SopB	HeLa cells	Increases the interaction of *Salmonella* with autophagosomes	Autophagy facilitates *Salmonella* replication in the cytosol of HeLa cells	(24)
**Affecting autophagy by subverting host cell homeostasis**					
*Salmonella* Typhimurium	SipB	BMDPM	Disrupts mitochondria to induce autophagy	Not applicable	(25)
*Shigella flexneri*	IcsB	MDAMC cells	Interacts with host cholesterol to evade autophagy	The absence of IcsB has no effect on bacterial survival up to 3 h of infection	(26)
*Burkholderia pseudomallei*	BopA	RAW 264.7 cells, MEFs, MDAMC cells	Interacts with host cholesterol to evade autophagy	Not applicable	(26, 27)
**Manipulating autophagy via unknown mechanisms**					
*Vibrio alginolyticus*	Unknown	Several mammalian cell lines	Activates autophagy via unknown mechanisms	Not applicable	(28)
*Pseudomonas aeruginosa*	Unknown	AEC line (A549 cells)	Unknown	Not applicable	(29)
*Yersinia enterocolitica*	Unknown	Murine J774A.1 macrophages	Suppresses autophagy via unknown mechanisms	Not applicable	(30)
**Escaping LC3-associated phagocytosis**					
*Shigella flexneri*	IcsB	HeLa cells, 293T cells and MEFs	Recruits the host protein Toca-1 to repress LC3 recruitment around these intracellular bacteria	The absence of IcsB has no effect on bacterial survival up to 3 h of infection	(11)
*Burkholderia pseudomallei*	BopA	RAW264.7 cells	Represses LC3 and LAMP1 recruitment via an unknown mechanism	*bopA* mutant bacteria show reduced intracellular survival	(7)

### Interference With Signaling or ATG Proteins Involved in Autophagy

*Shigella flexneri* effector IcsB, was recently found to repress the early recruitment of LC3 during infection (11). During early infection (40 min), IcsB recruits the host protein Toca-1 to intracellular *S. flexneri* to suppress the recruitment of LC3 and NDP52 around these intracellular bacteria. LC3 is a marker of autophagosomes, it is also present in LC3-associated phagocytosis. Therefore, this research suggests that IcsB manipulates Toca-1 to inhibit LC3-associated phagocytosis and/or LC3 recruitment to vacuolar membrane remnants early during infection (11). However, this study lacks supporting morphological observations.

In macrophages, SPI-2 (*Salmonella* pathogenicity island-2) T3SS is responsible for suppressing autophagy by actively manipulating the recruitment of focal adhesion kinase (FAK) to *Salmonella*-containing vacuoles (SCVs) and then stimulating the Akt-mTORC1 signaling pathway. However, the effector(s) that are responsible for this process remain unclear (12). Furthermore, the effector AWR5 from the plant pathogen *Ralstonia solanacearum* can also affect the mTOR signaling pathway to activate autophagy (13). Research indicates that AWR5, which is expressed heterologously in yeast, induces growth inhibition and autophagic flux. AWR5 may exert its function by inhibiting TORC1 upstream of PP2A directly or indirectly and thus promoting autophagy.

The effector SseF and SseG secreted by *Salmonella* Typhimurium can inhibit autophagy in host cells by the same autophagy blockade (14). Mechanistically, SseF and SseG impair autophagy initiation by directly interacting with the small GTPase Rab1A in the host cell. And the disruption of Rab1A signaling blocked the recruitment and activation of Unc-51–like autophagy-activating kinase 1 (ULK1) and decreased phosphatidylinositol 3-phosphate biogenesis, which ultimately suppress autophagosome formation.

*Salmonella* T3SS effector SopF was found to be a general xenophagy inhibitor without affecting canonical autophagy. Using *Salmonella* Typhimurium Δ*sopF*, the researchers identified the V-ATPase-ATG16L1 axis that mediates xenophagy initiation in HeLa cells. And SopF can target ATP6V0C for ADP-ribosylation on Gln124, thereby blocking bacterial autophagy and infection-induced recruitment of ATG16L1 by the V-ATPase (15).

We recently show that *Salmonella* Enteritidis effector AvrA can suppress autophagy (16). The AvrA protein is an effector that possesses acetyltransferase and deubiquitinase activity and inhibits the host c-Jun N-terminal kinase (JNK)/AP-1 and NF-κB signaling pathways; thus, AvrA inhibits host inflammatory responses and stabilizes intestinal tight junctions to the benefit of *Salmonella* survival (31, 32). We found that AvrA can inhibit autophagic responses by decreasing Beclin-1 protein levels, and this process occurs via JNK/c-Jun/AP-1 signaling pathway inhibition.

### Prevention of Recognition by Autophagy Mechanisms

Bacterial T3SS effectors can interfere with autophagy recognition mechanisms, thus avoiding bacterial killing in the host. IcsB from *S. flexneri* is one of the best-known effectors with this capability. IcsB competes with ATG5 binding to VirG (a bacterial surface protein), thereby masking the bacteria from recognition by autophagy mechanisms. Therefore, IcsB mutants are targeted for autophagy during multiplication in host cells infected with *S. flexneri* (18). Taken together, it suggests that the *S. flexneri* effector IcsB modulates LC3 recruitment around intracellular bacteria at the early stage of infection and inhibits autophagy late during infection (11, 18).

The *S. flexneri* T3SS effector IpaH1.4 is another example of preventing recognition by autophagy mechanisms (19). The E3 ligase LUBAC (liner ubiquitin chain assembly complex) can generate linear (M1-linked) polyubiquitin patches in the ubiquitin coat of intracellular bacteria, which recruit Optineurin and Nemo for xenophagy. In contrast, the effector protein IpaH1.4, a bacterial secreted E3 ubiquitin ligase 30, antagonizes the LUBAC-mediated accumulation of M1-linked ubiquitin chains on bacterial surfaces, as well as the recruitment of Optineurin and Nemo. Therefore, *S. flexneri* profoundly cripples LUBAC-dependent cellular defense mechanisms–xenophagy.

### Subversion of Autophagic Components for Bacterial Survival

T3SS effectors interact with autophagic components to interrupt autophagy. *Vibrio parahaemolyticus* VopQ is an effector that affects lysosomes. VopQ forms a gated ion channel in lysosomes to cause deacidification, thus disturbing autophagic flux. Moreover, VopQ binds directly to the V-ATPase V_o_ domain of lysosomes to block autophagosome-lysosome fusion (21–23).

It has been reported that *Salmonella* requires the RAB1 (the ras superfamily G-proteins-1)-mediated autophagy pathway for its survival (33). *Salmonella* Typhimurium SopB regulate this process to increases the interaction of *Salmonella* with autophagosomes for replication in HeLa cells. One possible reason for this finding is that SopB ubiquitination promotes the association of *Salmonella* and autophagosomes (24). Nevertheless, the detailed mechanism is still unclear.

### Escape From LC3-Associated Phagocytosis

LAP is not a member of the autophagy pathway. However, the autophagy marker LC3 protein participates in this process, which makes it hard to be ignored. We should distinguish autophagy from LAP in research work. For instance, effector IcsB, which may manipulate Toca-1 to inhibit LAP (11). The T3SS effector BopA also plays a role in preventing bacterial killing via LAP. Because the BopA mutants showed higher levels of colocalization with LC3 and the lysosomal marker LAMP1, suggesting enhanced elimination through LAP (7).

## Manipulating Autophagy via Bacterial Effectors From Other Secretion Systems

The effectors from other secretion systems of Gram-negative bacteria possess approaches for manipulating autophagy, as summarized in Table 2 and discussed in the following text (papers within 6 years).

**Table 2:** Strategies used by the effectors from T4SS, T6SS, and T7SS to manipulate autophagy.

**Bacteria**	**Effectors**	**Host model**	**Description**	**Bacterial survival**	**References**
**Interfering with signaling or proteins involved in autophagy**					
*Legionella pneumophila*	RavZ (T4SS)	HEK293 cells and MCF-7 cells	Cleaving LC3 off the membrane and modifying LC3 by its de-conjugating enzyme activity	Not applicable	(34, 35)
*Anaplasma phagocytophilum*	Ats-1 (T4SS)	THP-1 cells, RF/6A cells and *Beclin-1^+/−^* mice	Binds host Beclin-1 protein and hijacks Beclin-1-Atg14L autophagy initiation	Not applicable	(36)
*Vibrio parahaemolyticus*	VgrG2 (T6SS)	RAW264.7 cells	Possibly reduces the level of intracellular cAMP	Not applicable	(37)
*Ehrlichia chaffeensis*	Etf-1 (T4SS)	THP-1 cells, HEK293 cells and DH82 cells	Targets host RAB5, Beclin-1, VPS34, and autophagy - initiating PtdIns3K to ehrlichial inclusions to induce autophagy	*Ehrlichia chaffeensis* proliferation requires class III PtdIns3K activation and BECN1, and is enhanced by induction of autophagy with rapamycin	(6)
**Preventing recognition by autophagy mechanisms**					
*Bartonella quintana*	BepE (T4SS)	HeLa cells, HEK293 cells and HUVECs	Induces selective autophagy by conjugation with K63 poly-ubiquitin chain	Not applicable (But cells with BepE-induced autophagy are about 3-fold more effective at engulfing *Bartonella quintana* than cells with BepE-induced filopodia and membrane ruffles)	(38)
**Subverting autophagic components for bacterial survival**					
*Legionella pneumophila*	DrrA, LidA, RalF and LepB (T4SS)	Primary mouse macrophages	Interacts with RAB proteins to manipulate autophagosomal maturation	Not applicable	(39)
*Coxiella burnetii*	Cig2 (T4SS)	HeLa cells	Promotes the fusion of *Coxiella*-containing vacuoles with autophagosomes to maintain this vacuole in an autolysosomal stage of maturation	*Coxiella burnetii* is highly resistant to environmental stresses and is able to replicate in acidified lysosome-derived vacuoles	(40)
*Mycobacterium tuberculosis* (Note: this strain is not suitable for using Gram stain)	ESAT-6 (T7SS)	Human primary DCs	Impairs autophagosome-lysosome fusion	Not applicable	(41)
**Mediating autophagy by subverting host cell homeostasis**					
*Legionella pneumophila*	*Lp*Spl (T4SS)	HEK-293T cells and THP-1 macrophages	Inhibits autophagy by disrupting host sphingolipid biosynthesis	Not applicable	(42)
*Pseudomonas aeruginosa*	TplE (T6SS)	HeLa cells and HEK293T cells	Activates autophagy responses by subverting ER homeostasis	In intra- and inter-species competition studies show that the loss of *tplE* gave rise to a growth advantage of the recipient strain	(43)
**Manipulating autophagy via unknown mechanisms**					
*Brucella*	VceA (T4SS)	HPT-8 cells	Suppresses autophagy via unknown mechanisms	Not applicable	(44)
**Escaping LC3-associated phagocytosis**					
*Legionella* species	RavZ (T4SS)	HEK293 cells and MCF-7 cells	Cleaving LC3 off the membrane and modifying LC3 by its de-conjugating enzyme activity	Not applicable	(34, 45)
*Legionella pneumophila*	*Lp*Spl (T4SS)		May be responsible for inhibiting LAP	Not applicable	(42, 46)

### Interference With Signaling or ATG Proteins Involved in Autophagy

RavZ, is delivered by T4SS from *Legionella pneumophila*. RavZ can inhibit autophagy in HEK293 cells infected with *L. pneumophila* (34, 35). This protein uses its LIR motifs to bind to the LC3 protein and then extract LC3-PE (LC3- phosphatidylethanolamine) from the membrane of autophagosomes (35). RavZ hydrolyzes the amide bonds between glycine residues and aromatic residues at the carboxyl-terminal of the LC3 protein, using a catalytic mechanism similar to that of the Atg4. Thus, modified LC3 cannot be reconjugated by Atg7 and Atg3 in the process of autophagosome formation.

The protein VgrG2 from VpT6SS2 (T6SS-2 of *Vibrio parahaemolyticus*) induces autophagy by targeting the initial events of autophagic signaling (37). VgrG2 is a translocon of VpT6SS2. Heterogenous expression of VgrG2 increases LC3-II lipidation in macrophage cells and increases the accumulation of LC3-II in RAW264.7 cells treated with chloroquine (an inhibitor of autophagosome-lysosome fusion). Furthermore, VgrG2 mutants decrease the level of intracellular cAMP, which is necessary for the activation of the PRKA-AMPK-SIRT1 signaling pathway to induce autophagy in HUVECs treated with resveratrol (47). This finding suggests the possible role of targeting cAMP signaling in the VgrG2-mediated induction of autophagic responses (37).

A recent report has shown that *Ehrlichia chaffeensis* acquires nutrients from host cells by inducing RAB5-regulated autophagy via its T4SS-delivered effector Etf-1(6). Etf-1 interacts with RAB5, Beclin-1, VPS34, and autophagy-initiating PtdIns3K and is targeted to ehrlichial inclusions; through these mechanisms, Etf-1 induces autophagy to deliver host cytosolic nutrients for its replication while avoiding autophagic clearance (6).

### Prevention of Recognition by Autophagy Mechanisms

*Bartonella quintana* T4SS effector BepE was identified to induce selective autophagy. The researchers found that ectopic expression of BepE specifically induced punctate structures that colocalized with LC3-II in host cells. Further study showed that host cells utilize selective autophagy to confine and degrade BepE via poly-ubiquitin chain of K63 linkage conjugation (38).

### Subversion of Autophagic Components for Bacterial Survival

The *Coxiella burnetii* protein Cig2 is a T4SS effector that hijacks host autophagosomes. *Coxiella burnetii* is highly resistant to environmental stresses and is able to replicate in acidified lysosome-derived vacuoles. *Coxiella*-containing vacuoles (CCVs) are highly fusogenic with each other and with other organelles of the endocytic pathway; therefore, good-sized vacuole formation is promoted (48). The effector Cig2 can promote the fusion of CCVs with autophagosomes to maintain these vacuoles in an autolysosomal stage of maturation, thus promoting CCV homotypic fusion and influencing host infection tolerance in a moth model (40).

### Mediating Autophagy by Subverting Host Cell Homeostasis

A T4SS effector and a T6SS effector have been confirmed to affect autophagy by regulating other biological processes. The protein *Lp*Spl is translocated by the T4SS of *L. pneumophila*. *Lp*Spl has a high degree of similarity to eukaryotic sphingosine-1 phosphate lyase. Interestingly, cells infected with *Lp*Spl mutants have significantly greater LC3 recruitment than WT-infected cells, suggesting that *Lp*Spl is responsible for suppressing autophagy during infection. Together, these data indicate that *Lp*Spl inhibits autophagy by disrupting host sphingolipid biosynthesis. However, the complete mechanism has not been elucidated (42).

T6SS effector TplE from *Pseudomonas aeruginosa* is a Tle4 phospholipase family protein that possesses inter-bacterial killing capacity (49). Noteworthy, TplE targets and disrupts the host ER (endoplasmic reticulum) via its eukaryotic PGAP1-like domain. ER homeostasis perturbation can lead to the activation of the unfolded protein response, which acts as a potent trigger of autophagy (50). Therefore, TplE activates autophagy responses by subverting ER homeostasis (43).

### Manipulating Autophagy via Unknown Mechanisms

Effector VceA from *Brucella* T4SS is involved in host autophagic responses (44). As The Atg5, LC3-II, and Bcl-2 mRNA expression were significantly increased in the VceA mutant than the WT group. However, this study lacks the sufficient determining of autophagy process and the mechanism is still unclear.

### Escape From LC3-Associated Phagocytosis

T4SS of *Legionella* species has been reported to play a role in suppressing LAP (46). The T4SS effector RavZ from *L. pneumophila* strains can inhibit LAP via its capability to irreversibly deconjugate LC3, which has been previously described (34, 45). However, this LAP escape is not due solely to the effector RavZ in *L. pneumophila*; an additional strategy is likely utilized (46). One possible reason for this proposal is that the T4SS effector *Lp*Spl may be used to inhibit LAP. Sphingosine-1 phosphate lyase (*Lp*Spl)can decrease LC3 recruitment around *L. pneumophila* strains in macrophage cells (42).

## Conclusion, Limits, and Future Direction

From host side, the innate immunity and mucosal barriers play critical roles to maintain the autophagic responses (51, 52). From the bacterial side, effectors manipulate autophagy at different stages by using various strategies, including interfering with autophagy-initiating signaling, preventing the recognition of autophagy-involved proteins, subverting autophagy component homeostasis, manipulating the autophagy process (e.g., autophagosome maturation and autophagosome-lysosome fusion) and impacting other biological processes to affect autophagy. The research on effectors and autophagy has started to reveal basic features of autophagy manipulated by bacterial proteins for the benefit of bacterial survival and replication.

The progress in some field have shown better understanding of consequent host responses when autophagy is disturbed, such as killing host cells [SipB (25) and T3SS of *Vibrio alginolyticus* (28)], influencing host infection tolerance [Cig2 (40)], and escaping DC-mediated immune responses [ESAT-6 (41)]. Remarkably, bacteria could use multiple effectors (*Salmonella* and *Legionella*), and even two secretion systems (*Vibrio parahaemolyticus*), to mediate autophagy. Meanwhile, some effectors are versatile in manipulating autophagy (IcsB). Moreover, in the study of the effector *Lp*Spl, the author found that the effector RavZ is not present in all strains of *L. pneumophila*, suggesting that this strain employs other effector, namely *Lp*Spl (42), to inhibit autophagy. Therefore, determining whether one effector (even the partial function of one effector) is required for altering autophagic responses will help us to find novel effectors and better understanding of host-bacterial interactions.

The commonly used methods for exploring the effectors that are responsible for manipulating autophagy are: (a) deleting entire secretion systems or single genes and then analyzing the changes in autophagic responses in host cells infected with WT/mutant strains; (b) similarity searches to seek effectors that exhibit similar structures to host proteins, such as RavZ (35) and *Lp*Spl (42); and (c) investigating effectors that can interact with autophagy-involved proteins and then exploring the underlying mechanisms, such as Ats-1 (36) and Etf-1 (6). Most of these studies were done *in vitro* and still lack *in vivo* models to verify the physiological relevance of the studies. We would like to advocate the organoid system to study the host-microbial interactions (53). *In vivo*, acute and chronic infectious models will help us to understand into the short-term and long-term effects of bacterial survival and suppressed autophagy. When studying the interaction between a target and autophagy, one single autophagy marker is not sufficient for determining changes in autophagic responses. Determining multiple related proteins in autophagy, having morphological observations, and monitoring autophagic flux will support more information for judgment and help us to differentiate autophagy from other biological process, like LAP (54).

Noteworthy, the studies on effectors-autophagy interactions in host cells is not only to determine the effectors, that can affect the host autophagic responses, and the underlying molecular mechanism. Some excellent researches are progressing in the direction for understanding autophagy and its regulators. Like the study of effector VopQ, VopQ can bind directly to the V-ATPase V_o_ domain, which appears to play a key role in the regulation of autophagy through amino acid sensing, and even more directly, autophagosome-lysosome membrane fusion. Though the details is unclear, this study enlighten us to further elucidate the role of V-ATPase in autophagosome fusion with the lysosome (21, 23). Additionally, Xu et al. recently found *Salmonella* effector SopF is a xenophagy-specific inhibitor. By determining the target of *sopF*, they were able to identify the V-ATPase-ATG16L1 axis that mediates xenophagy initiation. Namely, upon infection, internalized bacteria cause damage to the residing vacuole, which is sensed by the vacuolar ATPase that then recruits ATG16L1 to initiate xenophagy. This study provides mechanistic insight into xenophagy recognition and initiation (15). It expands our knowledge to autophagic process, especially the crucial role of V-ATPase.

The causes of autophagy are numerous and complex. The mechanisms of effectors and autophagy have not been fully elucidated. Many important pathogenic Gram-negative bacteria still need to be tested. All the current studies focus on Gram-negative bacterial effectors, but Gram-positive and non-Gram stain bacterial effectors have been less attention (55). Moreover, the bacteria have abundant means/tools, not limited to effectors, to manipulate host autophagy. Most of these studies were focusing on uncovering the methods used by bacteria to inhibit autophagy, while ignoring the compensation of host cellular defense system. More studies are needed to understand the host cellular defense system in fighting back bacterial infection.

The physiological role of autophagy and its signaling mechanisms remain poorly understood. The study of interactions of bacterial effectors via pattern recognition receptors modulate the autophagy maybe uncover the underlying mechanisms. Overall, future directions could be focused on the following aspects in effectors-autophagy interactions to: (a) use various methods to determine multiple related proteins in autophagy, including morphological observations and monitoring autophagic flux to support accurate information about autophagic responses. Please refer to a comprehensive review of this subject (54); (b) understand the relationship between autophagy and immunological responses, which will uncover the link between autophagy and life activity. Most researchers pay attention to the effect of effectors to autophagic responses and the underlying cellular and molecular mechanism, but less attention to the immunological consequences of these affects; (c) study diverse immune and inflammatory signals modulate autophagy in host cell through pattern recognition receptors, such as toll-like receptors and nucleotide-binding oligomerization domain (NOD)-like receptors (56–59).

Studies on effectors-autophagy interactions in host cells will provide new insights into the pathogenic mechanisms of infections and inflammation. A better understanding of the mechanisms used by bacterial effectors to manipulate autophagy will help the study of mechanisms in immunity, drug design, and novel therapeutic approaches for infectious diseases and chronic inflammation.

## Author Contributions

JS designed the theme and topic and obtained research funds. YJ drafted the manuscript and organized the figure and tables. YJ and JS finalized the manuscript.

### Conflict of Interest

The authors declare that the research was conducted in the absence of any commercial or financial relationships that could be construed as a potential conflict of interest.

## Abbreviations

ATG protein: Autophagy-related protein
CCVs: *Coxiella*-containing vacuoles
DCs: Dendritic cells
ER: endoplasmic reticulum
JNK: c-Jun N-terminal kinase
LAP: LC3-associated phagocytosis
LUBAC: liner ubiquitin chain assembly complex
RAB proteins: the ras superfamily G-proteins
SCVs: *Salmonella*-containing vacuoles
SPI: *Salmonella* pathogenicity island
T3SS: type III secretion system
T4SS: type IV secretion system
T6SS: type VI secretion system
T7SS: type VII secretion system.
